# Editorial on case reports in pediatric immunology 2022

**DOI:** 10.3389/fped.2023.1242258

**Published:** 2023-08-16

**Authors:** Ankur Kumar Jindal

**Affiliations:** Allergy Immunology Unit, Department of Pediatrics, Advanced Pediatrics Centre, Postgraduate Institute of Medical Education and Research, Chandigarh, India

**Keywords:** Kawasaki disease (KD), multisystem inflammation syndrome, DiGeorge S, primary immune deficiencies, papillon lefevre syndrome, HLH—hemophagocytic lymphohistiocytosis

**Editorial on the Research Topic** Case reports in pediatric immunology 2022

Pediatric Immunology is a specialized field in pediatrics that deals with disorders of immune system. While most part of pediatric immunology is related to inborn errors of immunity [IEI, also known as primary immunodeficiency diseases (PIDs)], other diseases such as autoimmune rheumatic diseases, Kawasaki disease (KD) and multisystem inflammatory syndrome (MIS-C) associated with SARS-CoV-2 infection also forms a major part of this specialty. There have been several advances in the diagnosis and management of IEIs in last 2 decades. More than 450 different IEIs have been identified till date and every year new diseases are being discovered. The management of IEIs have evolved from immunoglobulin replacement to hematopoietic stem cell transplant, gene therapy and several novel targeted therapeutics. The research topic “Case Reports in Pediatric Immunology” is a collection of cases in Pediatric Immunology with diagnostic and therapeutic challenges.

Suzuki et al. reported a case of MIS-C that failed to respond to intravenous immunoglobulin (IVIg) and corticosteroids but showed a prompt response to cyclosporine. MIS-C has been reported to have several clinical and pathophysiological similarities with KD, a common childhood medium vessel vasculitis (1). The same group previously carried out a randomized controlled trial that reported the efficacy of cyclosporine in combination with IVIg in patients with KD who were predicted to be non-responsive to IVIg alone. The combination of IVIg and cyclosporine resulted in better coronary artery outcomes in these patients.

Basu et al. reported successful use of corticosteroids for liver abscess in patients with Papillon-Lefèvre syndrome. This disorder is characterized by a defect in neutrophil extracellular trap formation in response to reactive oxygen species. Although, the role of corticosteroids in management of liver abscess is well established for patients with chronic granulomatous disease (2), this is for the first time that its role has also been evaluated for management of liver abscess in Papillon-Lefèvre syndrome.

Poplonyk et al. reported a case of cardio-facio-cutaneous syndrome, which is a group of RASopathies (3), caused by a novel germline mutation in *MAP2K1* gene. PIDs with syndromic features is a special group disorders that are clinically and pathophysiologically heterogeneous. A high index of suspicion is needed to diagnose these disorders early. In children who have syndromic features and recurrent infections, the infections may commonly be attributed to neurological dysfunction, aspiration or to the cardiac defects. However, it is important to evaluate for underlying immune abnormalities as this may change the management in these cases.

A proportion of patients with DiGeorge syndrome have an underlying immunodeficiency. Autoimmunity is being increasingly recognized in these cases (4). Gu et al. report a case of successful use of sirolimus in a patient with DiGeorge syndrome who presented with autoimmune lymphoproliferative syndrome (ALPS) like features. Autoimmune lymphoproliferative syndrome is a well-defined PID and role of sirolimus in these patients is established. However, several PIDs may also present with ALPS like phenotype. It is important to identify this phenotype so that more targeted therapies such as sirolimus can be used for these patients.

The newborn screening for severe combined immunodeficiency (SCID) has revolutionized this field and this potentially fatal disease is now being recognized early in life and curative treatment is being offered. Universal newborn screening for SCID is now being offered in all parts of the United States, several European and a few Asian countries. Vasco et al. report a case of GATA2 deficiency that was diagnosed using new born screening for SCID. GATA-2 deficiency has a wide spectrum of clinical phenotype and immunodeficiency is an important component of this syndrome.

Genetic sequencing has provided novel insights into the pathogenesis of PIDs (5). Several new genetic defects and novel variants are being identified in PIDs. Zhu et al. identified a case of Capping protein regulator and myosin 1 linker 2 (CARMIL2) deficiency using whole exome sequencing. CARMIL2 deficiency is a combined immunodeficiency with a broad clinical phenotype. Disseminated warts, recurrent respiratory infections and eczema are important clinical manifestations.

Familial hemophagocytic lymphohistiocytosis (HLH) is a potentially life-threatening disorder caused by defect in genes that are important for perforin synthesis and natural killer or cytotoxic T cell degranulation. Familial HLH may have varied clinical presentation. Central nervous system (CNS) involvement, especially when it is isolated, pose a diagnostic challenge. Yoshida et al. reported a case of familial HLH caused by *UNC13D* gene mutation who presented with unusual clinical presentation, i.e., cerebellar swelling and hydrocephalus.

Sgrulletti et al. report a case that shows the diagnostic conundrum between primary and secondary immunodeficiency. Patients with PIDs are predisposed to develop autoimmune manifestations and malignancy. At times, these may be the first and only clinical presentation. Often unaware of this aspect of PIDs, the treating physician may initiate immunosuppressive therapy including rituximab in a few cases of autoimmunity or malignancy. It may be extremely difficult to differentiate the hypogammaglobulinemia caused by use of immunosuppressant drugs from primary hypogammaglobulinemia. It is suggested to perform immunoglobulin levels in all patients before initiating rituximab. Genetic testing may be helpful in these cases.

Sun et al. report an adolescent girl with autoimmune polyglandular syndrome type III C + D who presented with Hashimoto's thyroiditis, vitiligo, anemia, pituitary hyperplasia, and lupus nephritis. This is an unusual presentation in a child. The pathophysiological mechanisms and immune basis are not clear.

Chimeric antigen receptor (CAR) T-cell therapy is an upcoming management strategy for several disorders especially malignancy. Hypogammaglobulinemia is a common complication of CAR T-cell therapy and this complication is more common in children. As a result, patients may be at risk of unusual infections following CAR T-cell therapy. Sanders et al. report a case of B-cell acute lymphoblastic leukemia (B-ALL) who received CAR T-cell therapy and developed hypogammaglobulinemia requiring immunoglobulin replacement therapy. He subsequently developed SARs-CoV-2 infection requiring a combination of antimicrobials.
